# Low Intratumoral CD200 Protein Expression in Primary Merkel Cell Carcinoma Is a Strong Predictor for Disease Relapse

**DOI:** 10.3390/cancers17050822

**Published:** 2025-02-27

**Authors:** Thilo Gambichler, Sophia Girke, Nessr Abu Rached, Laura Susok, Jürgen C. Becker, Hans-Joachim Schulze, Tobias Hirsch, Maximilian Kückelhaus, Sascha Wellenbrock

**Affiliations:** 1 Skin Cancer Center, Department of Dermatology, Ruhr-University Bochum, 44787 Bochum, Germany; sophia.girke@rub.de (S.G.); n.aburached@klinikum-bochum.de (N.A.R.); laura.susok@klinikumdo.de (L.S.); 2 Department of Dermatology, Klinikum Dortmund gGmbH, University Witten/Herdecke, 44137 Dortmund, Germany; 3 Department of Dermatology, Christian Hospital Unna, 59423 Unna, Germany; 4 Translational Skin Cancer Research, DKTK Partner Site Essen/Düsseldorf, West German Cancer Center, Department of Dermatology, University Duisburg-Essen, 45122 Essen, Germany; j.becker@dkfz-heidelberg.de; 5 German Cancer Research Center (DKFZ), 69120 Heidelberg, Germany; 6 Department of Dermatology, Fachklinik Hornheide, 48149 Munster, Germany; schulze@fachklinik-hornheide.de; 7 Department of Plastic, Reconstructive and Aesthetic Surgery, Hand Surgery, Fachklinik Hornheide, 48157 Munster, Germany; tobias.hirsch@fachklinik-hornheide.de (T.H.); maximilian.kueckelhaus@fachklinik-hornheide.de (M.K.); sascha.wellenbrock@yahoo.com (S.W.); 8 Department of Plastic Surgery, University Hospital Munster, 48149 Munster, Germany; 9 Department of Plastic and Reconstructive Surgery, Institute for Musculoskeletal Medicine, University Hospital Munster, 48149 Munster, Germany

**Keywords:** Merkel cell carcinoma, immune checkpoints, CD200/CC200R-signaling, OX-2, immunotherapy

## Abstract

Background: Merkel cell carcinoma (MCC) is a rare and frequently fatal form of skin cancer. Apart from Programmed Cell Death Protein 1 (PD-1)/Programmed Death-Ligand 1 (PD-L1) signaling, there is a lack of knowledge regarding other immune checkpoint molecules. Recent studies have observed elevated glycoprotein CD200 (also known as OX-2) mRNA expression in in different types of tumors, with CD200R-expressing myeloid cells present in the tumor microenvironment. However, the potential role of the CD200/CD200 axis as an additional checkpoint modulator remains widely unexplored. The aim of this study was to determine the intratumoral protein expression of CD200 as well as CD200R in a larger cohort of MCC patients and to correlate the expression levels with patients’ outcomes. Methods: In this multicenter study, we investigated 68 patients with MCC (68 primary tumors and 15 corresponding metastases). Immunohistochemistry (IHC) was performed for CD200 as well as CD200R. Digital quantification and analysis of IHC were performed using QuPath-0.2.3. Results: CD200 and CD200R expression was observed in 100% of cases. Univariate analysis revealed that low CD200 expression in primary tumors (*p* = 0.0007, HR 9.35), male sex (*p* = 0.045, HR 2.41), and immunosuppression (*p* = 0.0031, HR 6.36) were significantly associated with MCC relapse. Low CD200 expression was also linked to prior immune checkpoint inhibitors (ICI) and/or chemotherapy treatment (*p* = 0.037). Multivariable analysis confirmed that low CD200 expression (*p* = 0.0012, HR 5.25) and immunosuppression (*p* = 0.0056, HR 4.11) were independent predictors of MCC relapse. Conclusions: Expression of CD200/CD200R proteins is very high in MCC and may thus be of diagnostic value. More importantly, low intratumoral CD200 protein expression in primary MCC represents a robust independent predictor of MCC relapse.

## Simple Summary

Merkel cell carcinoma (MCC) is a rare and aggressive skin cancer. This study investigated the role of the immune-related proteins CD200 and CD200R in MCC. All tumors showed high expression of these proteins, but multivariable analysis revealed two key factors independently associated with a higher risk of cancer relapse: low CD200 expression and patient immunosuppression. Specifically, patients with low CD200 expression were over five times more likely to experience relapse, while immunosuppressed individuals had a fourfold increased risk. These results highlight the potential of CD200 as a predictive marker for MCC recurrence, which could help guide future treatment strategies.

## 1. Introduction

Merkel cell carcinoma (MCC) is a rare and frequently fatal form of skin cancer [1,2,3]. Stang et al. [4] recently analyzed data from the cancer registry of North Rhine-Westphalia, Germany, during 2008–2021, covering a population of 18 million. They observed that the age-standardized incidence of MCC was 5.2 (men) and 3.8 (women) per million person-years. The 5-year relative survival was 58.8% (men) and 70.7% (women) [4]. Hence, the biological behavior of MCC is highly aggressive with high rates of local recurrences and regional lymph node and distant metastasis. Major risk factors for MCC are chronic UV exposure, high age, and immune suppression [1,2,3]. In recent years, the treatment landscape for advanced MCC has significantly evolved. Besides radiation therapy and chemotherapy as previous standard treatments for advanced MCC, targeted therapies and vaccine platforms are now being investigated, including those targeting the Merkel cell polyomavirus (MCPyV) and its associated oncogenic pathways being explored in clinical trials [1,2,3]. Despite all alternatives, immune checkpoint inhibitors (ICIs) in the treatment of advanced MCC, such as anti-PD-1 inhibitors (pembrolizumab, nivolumab, and retifanlimab) and the anti-PD-L1 inhibitor avelumab, have revolutionized therapies, as they have dramatically improved outcomes for patients with advanced MCC, offering durable responses and better overall survival compared to traditional chemotherapy. Hence, ICI is considered the first-line approach for metastatic MCC [3,5,6,7,8]. ICIs in the metastatic setting of MCC are frequently associated with durable response rates (about 70%) and a 3-year overall survival of approximately 65% [7,8]. Nevertheless, about 50% of patients do not respond to ICIs. Apart from PD-1/PD-L1 signaling, there is a lack of knowledge regarding other immune checkpoint molecules, including the CD200/CD200R axis, concerning MCC evolution and prognosis [9,10,11].

The CD200/CD200R signaling pathway is a key regulator of immune responses, known for its immunosuppressive effects in various physiological and pathological contexts, including malignancies such as melanoma. CD200 is a cell surface glycoprotein that binds to its receptor, CD200R, primarily expressed on myeloid and lymphoid cells and, in case of malignancies, also on tumor cells. This interaction may inhibit pro-inflammatory responses and promotes immune tolerance, often exploited by tumors to evade immune detection [12,13]. In melanoma and other cancers, CD200 overexpression has been linked to immune evasion. Tumor cells expressing CD200 can suppress macrophage and dendritic cell activation, reduce T-cell cytotoxicity, and foster an immunosuppressive tumor microenvironment [14,15,16,17]. Elevated CD200 expression often correlates with worse prognosis and increased metastatic potential in melanoma [18,19]. While the immunosuppressive and tumor-promoting effects of CD200/CD200R are well documented, evidence suggests this pathway may not always favor tumor progression. Some studies have proposed that CD200 signaling might limit chronic inflammation, which can otherwise create a pro-tumorigenic environment [20,21,22]. For instance, reduced inflammation might prevent the recruitment of myeloid-derived suppressor cells and other cells that support tumor growth [23]. Furthermore, CD200 expression on tumor-infiltrating immune cells could help modulate excessive immune activation, thereby reducing tissue damage and maintaining immune homeostasis. Indeed, the dual role of CD200/CD200R signaling complicates both its pathophysiological significance in cancers and therapeutic targeting [20]. With regard to MCC protein and mRNA, expression of CD200 has been studied only in two investigations [24,25]. Using immunohistochemistry, Love et al. [24] investigated a panel of different neuroendocrine neoplasms, including MCC, which expressed CD200 in 84% of cases. Moreover, they concluded that the expression of CD200 in MCC merits further study to determine if CD200 plays a role in the biology of the disease, if CD200 represents a potential therapeutic target, or if loss of CD200 affects prognosis [24]. Gaiser et al. [25] previously evaluated CD200 expression by immunohistochemistry in primary tumors and MCC metastases. Gaiser et al. [25] found no correlation between CD200 expression levels and MCC tumor stage at diagnosis, progression-free survival, and MCC-specific survival. However, Gaiser et al. [25] observed that intravenous administration of blocking anti-CD200 antibody to MCC xenograft mice revealed specific targeting of the drug to the tumor and thus concluded this treatment may provide a novel immunotherapeutic approach for MCC independent of PD-1/PD-L1 inhibition. CD200R was not addressed in the studies of Love et al. [24] and Gaiser et al. [25].

Hence, the aim of this exploratory study was to determine the intratumoral protein expression of CD200 as well as CD200R in a larger cohort of MCC patients and to correlate the expression levels with patients’ outcomes.

## 2. Materials and Methods

### 2.1. Patients

According to accepted histopathological and immunohistochemical criteria, the diagnosis was confirmed by two experienced dermato-histopathologists. Complete clinical work-up and follow-up was performed according to the current national guidelines [1,2]. MCC restaging was performed in accordance with the 8th edition of the AJCC guideline [1,26]. Clinical data were collected both by chart review as well as contacting patients, relatives, resident practitioners, and dermatologists to retrieve missing data. The study was approved by the local ethics review board of the Medical Faculty of the Ruhr-University Bochum (#16-5985).

### 2.2. Analysis of Merkel Cell Polyomavirus (MCPyV) in FFPE Tissue

MCPyV viral load determination was performed on a LightCycler 480 Real Time PCR System (Roche, Grenzach, Germany) as described previously [27]. Briefly, MCPyV DNA load was determined by quantitative real-time PCR using MCPyV-specific LT3-primers and a locked nucleic acid probe binding to the N-terminal part of the large T-antigen gene (TAg). MCPyV DNA load was expressed as MCPyV DNA copies per betaglobin-gene copy.

### 2.3. Immunohistochemistry of MCC Tumor Samples

We studied the primary tumors of 68 patients and corresponding metastases of 15 patients. Immunohistochemistry was performed in accordance with the manufacturer’s recommendations and previous papers [28]. Briefly, sections (4 µm) of formalin-fixed (4% buffered), paraffin-embedded tissues were dried overnight at 37 °C, deparaffinized in Rotihistol (Carl Roth, Karlsruhe, Germany), and subsequently hydrated through a graded alcohol series. For immunostaining, we used primary antibodies against CD200 (OX2, rabbit, polyclonal, and ab254193; Abcam, Cambridge, UK) and CD200R (rabbit, monoclonal, and ab23294; Abcam). Staining was performed using the Dako Link 48 autostainer (Dako, Santa Clara, CA, USA). Visualization was performed using the Dako REAL^TM^ Detection System, Alkaline Phosphatase/RED, Rabbit/Mouse (K5005, Dako Agilent; Santa Clara, CA, USA) according to the manufacturer’s protocol. For nuclear counterstaining, specimens were incubated in hematoxylin (S202084, Dako Agilent) for 1 min followed by a 5 min incubation in tap water. Finally, samples were processed through a series of ascending alcohol concentrations and mounted with Entellan (Merck, Darmstadt, Germany).

### 2.4. Microscopic Evaluation

For microscopic analysis, stained slides were scanned at 20× magnification using the Nanozoomer Whole Slide Scanner from Hamamatsu (Hamamatsu, Herrsching am Ammersee, Germany). The images were evaluated by using the viewer software NDP.view2 (Hamamatsu Photonics, Germany). Digital quantification and analysis were performed using QuPath-0.2.3 (Queen’s University Belfast, Belfast, Northern Ireland, UK), an open-source bioimage analysis software [29]. Automated cell counting of positive and negative cells was then performed over the predefined intratumoral area of interest. The data were analyzed by relating the total number of positive cells to the number of cells detected in a given compartment. Staining intensity was also evaluated (0 = none; 1 = slight; 2 = moderate; 3 = strong) and multiplied with the percentage of positive cells, resulting in a totalized H-score ranging from 0 to 300 [30]. Membranous staining of tumor cells and tumor-infiltrating cells was predominantly evaluated.

### 2.5. Statistics

Data analysis was performed using the statistical package MedCalc Software version 23.02. (MedCalc Software, Ostend, Belgium). Distribution of data was assessed by the D’Agostino-Pearson test. Normally distributed data are expressed as mean and standard deviation (SD) and non-normally distributed data as medians and range. Where appropriate, data were analyzed using the Wilcoxon test, Kendall’s tau procedure, chi-square test, Kaplan–Meier curves, and ROC analysis. Multivariable testing was performed using a Cox proportional-hazards regression model only including independent variables that were statistically significant on univariable analysis. Hazard ratios (HR) and 95% confidence intervals (CI) are reported. *p*-values of <0.05 were considered significant.

## 3. Results

### 3.1. Patients’ Characteristics

Sixty-eight patients [median age: 76.2 years (50–94); 34 males, 34 females)] were studied for whom formalin-fixed paraffin-embedded tumor tissue was available for immunohistochemistry. In 34 of 68 (50%) patients, primary tumors were observed in the high-risk region (head/neck). Data on MCPyV were available only in 18 patients (26.5%). At time of diagnosis including the first complete work-up, 19 of 68 patients (37.5%) were in stage I, 24 (35.3%) in stage II, 21 (30.9%) in stage III, and 4 of 68 (5.9%) in stage IV according to the 8th edition of the American Joint Committee on Cancer staging system for MCC [13]. In total, 14 of 68 (20.6%) patients were immunosuppressed. Treatments for advanced patients in the course of the disease included ICI 11 (16.2%), chemotherapy 7 (10.3%), and other modalities 15 (22%, Table 1).

### 3.2. Expression of CD200/CD200R

**Figure 1:**
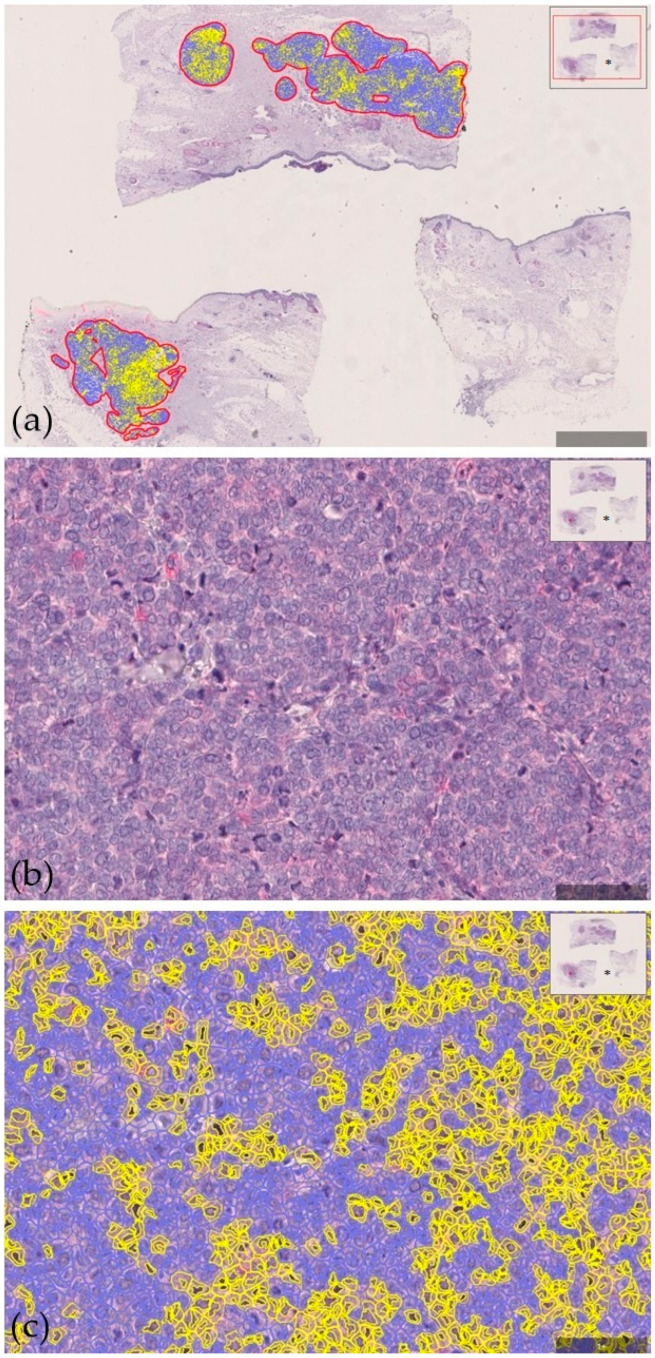
Low intratumoral CD200 protein expression in a 76-year-old male with Merkel cell carcinoma who experienced disease relapse three months after first diagnosis. Specific staining of cells with CD200 expression, oversight view. QuPath-0.2.3 marks (**a**); standard immunohistochemistry, 100× magnification (**b**); specific staining of cells with CD200 expression, 100× magnification; QuPath-0.2.3 marks (**c**); * oversight of the scanned specimen (**a**–**c**).

**Figure 2:**
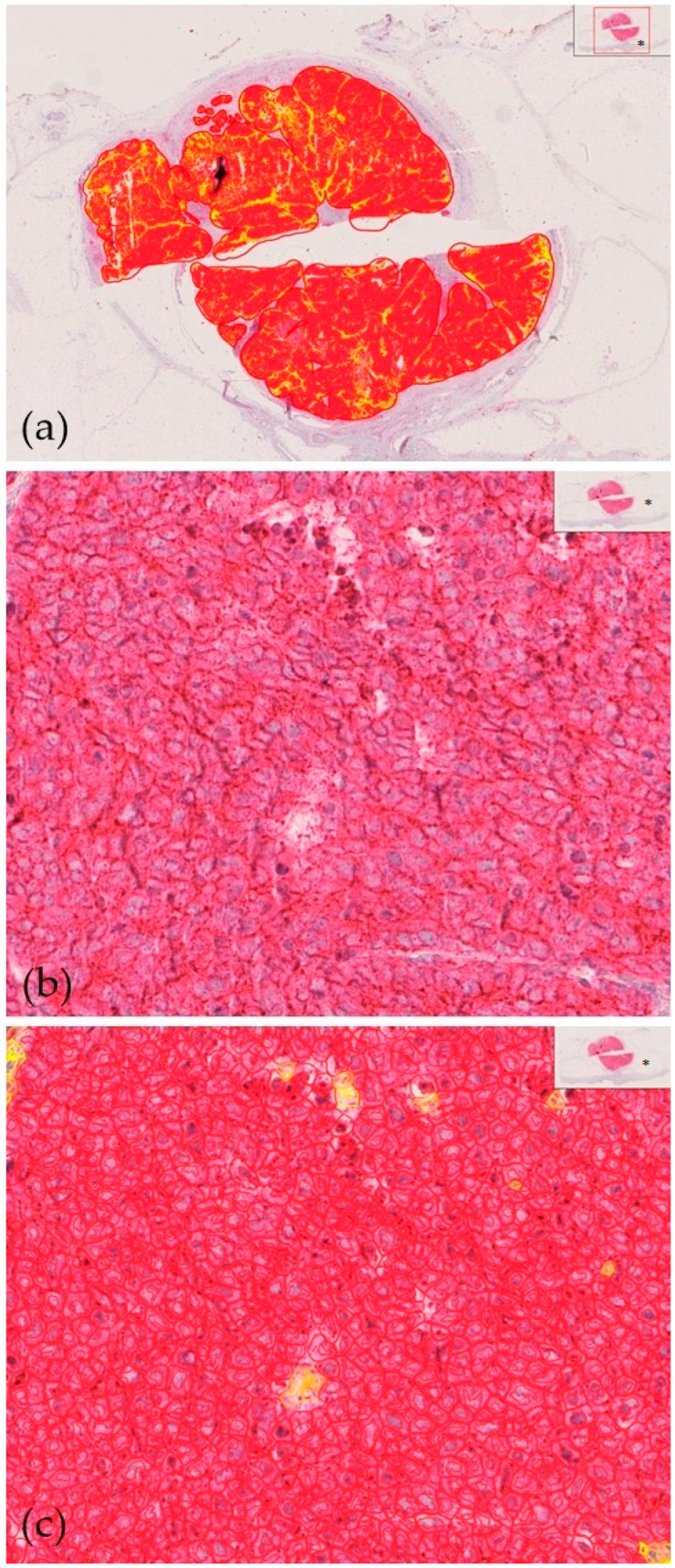
High intratumoral CD200 protein expression in a 69-year-old female with Merkel cell carcinoma who did not experience a disease relapse within a 43-month follow-up after first diagnosis. Specific staining of cells with CD200 expression, oversight view. QuPath-0.2.3 marks (**a**); standard immunohistochemistry, 100× magnification (**b**); specific staining of cells with CD200 expression, 100× magnification; QuPath-0.2.3 marks (**c**); * oversight of the scanned specimen (**a**–**c**).

**Figure 3:**
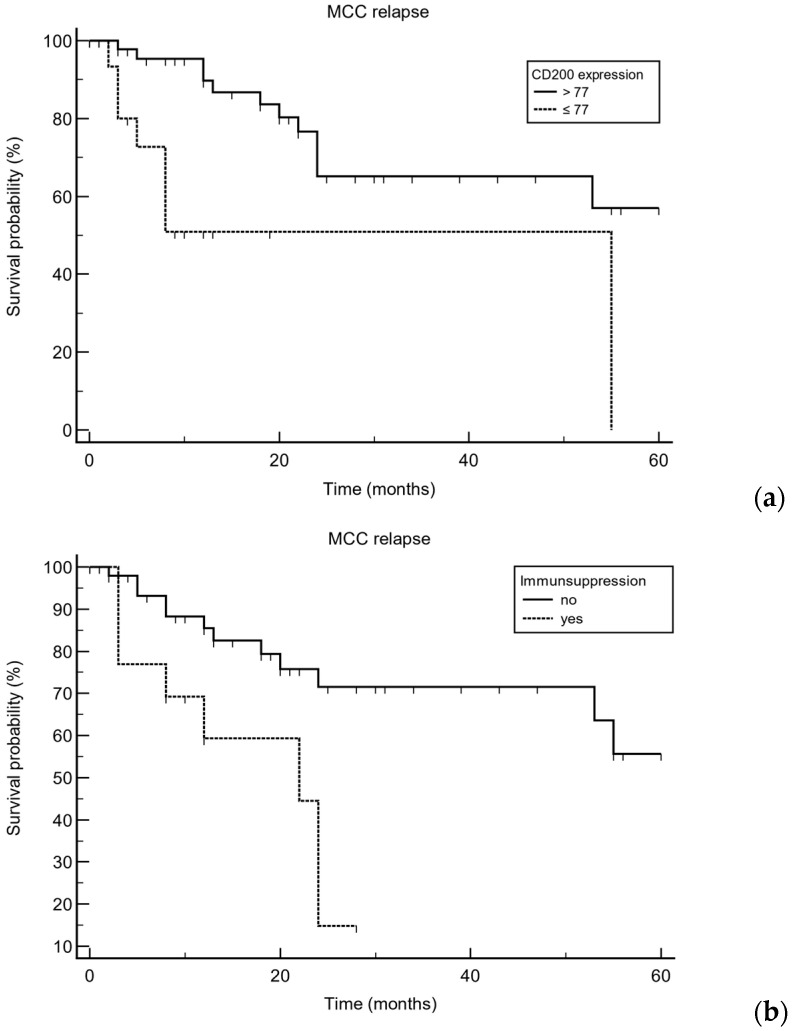
Kaplan–Meier curves showing that low expression of CD200 (≤77, (**a**)) in primary Merkel cell carcinoma (MCC) is associated with MCC relapse (*p* = 0.0007, HR 9.35, 95% CI 2.56 to 34.17). Moreover, the presence of immunosuppression (**b**) is also associated with MCC relapse (*p* = 0.0031, HR 6.36, 95% CI 1.87 to 21.65).

As shown in Table 2, microscopic evaluation revealed high median (range) expression for CD200 [171.5 (3–284)] and CD200R [167 (53–243)]. CD200 and CD200R expression did not differ between primary tumors and metastases (*p* = 0.68 and *p* = 0.71, respectively). CD200 as well as CD200R expression was observed in 100% of cases. Only in primary tumors were CD200 and CD200R significantly correlated with each other (*r* = 0.27, *p* = 0.0028). On univariable analysis, we observed that MCC relapse was significantly associated with low CD200 expression in primary tumors (H-score ≤ 77, 25th percentile of CD200 expression, *p* = 0.0007, HR 9.35, 95% CI 2.56 to 34.17), male sex (*p* = 0.045, HR 2.41, 95% CI 1.009 to 5.76), and immunosuppression (*p* = 0.0031, HR 6.36, 95% CI 1.87 to 21.65; Figure 1, Figure 2 and Figure 3). Low CD200 protein expression in primary tumors was also associated with administration of ICI and/or chemotherapy during the course of disease (*p* = 0.037). On multivariable analysis, low CD200 expression was significantly correlated with MCC relapse (*p* = 0.0012, HR 5.25, 95% CI 1.93 to 14.29). Moreover, the presence of immunosuppression was an independent predictor for MCC relapse (*p* = 0.0056) as indicated by an HR of 4.11 (95% CI 1.51 to 11.19). However, male sex did not remain significant in the regression model. Age at MCC diagnosis was the only significant predictor of MCC-specific death (*p* = 0.0040, HR 1.086, 95% CI 1.026 to 1.149). CD200 and CD200R protein expression in primary tumors did not correlate with stage of disease (*p* = 0.43 and *p* = 0.49, respectively). Unlike CD200 expression in primary MCC, the expression profiles of CD200R did not correlate with clinical outcome measures such as MCC relapse or MCC-specific death (*p* > 0.05).

### 3.3. Patients’ Treatment and Outcome

Patients were treated in accordance with German guidelines [1]. Hence, primary tumors were fully excised with a safety margin of 1–2 cm depending on tumor diameter and anatomic site. The patients received adjuvant radiotherapy in the tumor bed and draining lymph node region after having undergone sentinel lymph node dissection. In case of lymph node metastases, complete lymph node dissection was performed. Most patients of advanced stage received radiotherapy, electrochemotherapy, and systemic chemotherapy (e.g., carboplatin and etoposide). Since many patients were treated in the pre-ICI era, only eleven patients of advanced stages actually received ICI. Within a median follow-up period of 12 months (3–113 months), 22 of 68 patients (32.4%) experienced a disease relapse, and 22 (32.4%) patients died from MCC within a median follow-up period of 18 months (3–133 months; Table 1).

## 4. Discussion

CD200 expression was previously investigated in two studies [24,25]. Using immunohistochemistry, Love et al. [25] investigated a panel of different neuroendocrine neoplasms, including 149 MCC. The latter expressed CD200 in 125 cases (84%), with a diffuse membranous staining pattern of neoplastic cells. However, it was not clear whether they investigated primary tumor and/or metastases. Moreover, they did not correlate CD200 expression with clinical outcome measures. Despite this finding, they concluded that CD200 evaluation will likely not become a routine part of the diagnosis of MCC, which already has a well-defined immunoprofile of paranuclear, dot-like CK20 positivity and lack of CK7 expression [1,2,3]. Love et al. [25] further concluded that the expression of CD200 in MCC merits further studies to determine whether CD200 plays a role in the biology of the disease, if CD200 represents a potential therapeutic target, or if loss of CD200 affects prognosis. Moreover, Gaiser et al. [24] previously evaluated CD200 expression by immunohistochemistry in 53 primary tumors and 35 MCC metastases (local recurrence, lymph node metastases, in-transit metastases, and distant metastases). Overall, 84 of 88 analyzable MCC specimens (95.5%) showed CD200 staining, including 51 of 53 primary tumors (96.2%) and 33 of 35 metastases (94.3%). Overall, 59 of 61 patients (96.7%) had CD200 staining in their tumors [24]. Using 59 cases with analyzable MCC tumor tissue from the date of diagnosis, Gaiser et al. [25] found no correlation between CD200 expression levels and MCC tumor stage at diagnosis. Based on Kaplan–Meier estimates, neither progression-free survival nor MCC-specific survival differed based on CD200 expression levels. Moreover, Gaiser et al. [25] observed that intravenous administration of blocking anti-CD200 antibody to MCC xenograft mice revealed specific targeting of the drug to the tumor and thus concluded this treatment may provide a novel immunotherapeutic approach for MCC independent of PD-1/PD-L1 inhibition.

Consistent with the findings of Love et al. [25] and Gaiser et al. [24], we observed a notably high expression of CD200 and CD200R proteins in 100% of cases. Therefore, we propose that these markers could serve as supplementary immunohistochemical tools for distinguishing MCC from other malignancies in specific situations. Similar to the data reported by Gaiser et al. [25], we did not obsereve a difference between CD200 protein expression in primary tumors and metastases. Moreover, we did not identify a correlation between CD200 expression at diagnosis and stage of MCC, indicating that CD200/CD200R signaling is likely not significantly involved in development and progression of MCC. In the present study, we observed that MCC relapse was significantly associated with low CD200 expression in primary tumors, male sex, and immunosuppression. Male sex and immunosuppression are well-documented risk factors for poor prognosis of MCC patients [1,2,3,4]. More importantly, besides the presence of immunosuppression, low CD200 expression was a significant predictor of MCC relapse on multivariable analysis. Accordingly, low CD200 protein expression in primary tumors was associated with the administration of ICI and/or chemotherapy in the course of the disease. In line with previous data, age at MCC diagnosis was the only significant predictor in our study.

In the present study, the expression profiles of CD200R did not correlate with clinical outcome measures such as MCC relapse or death. Interestingly, we recently showed on Cox proportional-hazards regression analyses that higher CD200R protein expression was a significant predictor of objective response to ICI [22]. More importantly, low CD200R protein expression was the only significant independent predictor for progression-free survival and melanoma-specific survival [22]. Although MCC and melanoma are distinct tumor entities with different underlying pathobiology, how do our data align with the previously described pro-cancer effects of the CD200/CD200R signaling pathway? Indeed, the CD200/CD200R signaling pathway plays an etiological role in the survival and metastasis of numerous cancers primarily through suppressive effects on anti-cancer immune surveillance [31]. CD200/CD200R interactions may be much more complex or even bidirectional in malignancies. Liu et al. [32] further investigated the role of CD200R signaling in tumor progression and metastasis using CD200R^−/−^ mice. They observed that CD200R^−/−^ mice showed enhanced growth of CD200+ but not CD200- B16 tumors. Interestingly, CD200R^−/−^ mice receiving CD200+ B16 cancer cells showed striking tumor growth in multiple organs, while the growth of the same tumor in wild-type mice was limited. Hence, Liu et al. [32] detected a critical role of CD200R signaling in limiting tumor progression and metastasis of CD200+ tumors. Recently, they observed similar results in a melanoma mouse model [13]. Furthermore, Erin et al. [20] assessed the role of CD200/CD200R1 signaling in the progression and metastasis of 4THM murine-breast carcinoma using CD200 transgenic (CD200tg) and CD200R1 knock-out (CD200R^−/−^) mice. They found that CD200 overexpression in the host was associated with diminished primary tumor growth and metastasis, while loss of CD200R1 expression by host cells was linked to increased visceral metastasis [20]. Loss of CD200R1 expression resulted in decreased tumor-infiltrating CD8+ cells and enhanced the secretion of inflammatory cytokines [20]. While blocking the pathway could enhance anti-tumor immunity, it might also disrupt protective mechanisms that prevent inflammation-driven tumorigenesis. Together, the CD200/CD200R axis represents a promising but complex therapeutic target in malignancies. Its tumor-promoting and potentially tumor-suppressive effects highlight the need for a nuanced understanding of its role in cancer. Strategies aimed at modulating this pathway must carefully balance the benefits of enhancing immune responses against the risks of exacerbating inflammatory processes [33].

Blockade of CD200/CD200R interactions using monoclonal antibodies like samalizumab has shown promise in restoring anti-tumor immunity, particularly in hematologic malignancies. Furthermore, anti-CD200R agonists have proven effective in reshaping the tumor microenvironment and improving the anti-tumor effects of other immunotherapies. Hence, the interaction between CD200 and other immune checkpoint molecules has also garnered attention, with evidence suggesting that CD200 expression can influence the efficacy of ICI. Understanding the intricate network of checkpoint molecules and their impact on immunotherapy response remains a critical area of research [33]. Together, the motivation for this study stems from the pressing need to enhance diagnostic accuracy and prognostic assessment in rare and aggressive skin cancers such as MCC. Recent advances have demonstrated significant potential in improving the detection, classification, and prognostication of various skin cancers [34,35].

While this study provides valuable insights into the role of CD200/CD200R expression in MCC, several limitations must be considered. Firstly, the sample size of 68 patients, although relatively large for MCC studies, remains modest and may limit the generalizability of the findings. Additionally, the retrospective nature of the study introduces potential biases related to patient selection and data collection, which could be addressed by prospective studies. Secondly, while immunohistochemistry was used to assess protein expression, its inherent limitations, such as variability in antibody specificity and subjective interpretation, could affect reproducibility, despite using digital quantification with QuPath-0.2.3. Thirdly, although the study controlled for clinical variables like sex, immunosuppression, and treatment, it may not have accounted for confounding factors such as other clinical or molecular markers influencing MCC progression. Lastly, the study focused on protein expression without exploring gene expression analyses and functional mechanisms. Future studies that investigate how CD200 modulates immune responses and tumor progression in MCC would offer deeper insights. Validation using independent cohorts would also strengthen the findings, ensuring broader applicability. Indeed, from the clinical point of view, larger, prospective multicenter studies are needed to confirm the results of our exploratory study.

## 5. Conclusions

This study emphasizes the potential of CD200 as a prognostic biomarker in Merkel cell carcinoma (MCC). Our results show that CD200 and CD200R proteins are highly expressed in MCC. Specifically, low CD200 expression in primary tumors is strongly linked to an increased risk of MCC relapse, highlighting its potential as an independent predictor of disease outcome. While CD200R expression did not correlate with clinical factors, the widespread expression of both CD200 and CD200R in MCC suggests their diagnostic value. These findings advocate for further exploration of the CD200/CD200R axis as a therapeutic target in MCC, especially in relation to immunosuppression and treatments like immune checkpoint inhibitors.

## Figures and Tables

**Table 1:** Clinical characteristics and results of CD200/CD200R protein expression analysis in patients with Merkel cell carcinoma (MCC, *n* = 68).

Parameters	Data
**Age at diagnosis** *	
Years	76.2 (50–94)
**Gender**	
Male	34 (50%)
female	34 (50%)
**Primary MCC localization**	
Head/neck	34 (50%)
Upper extremities	23 (33.8%)
Lower extremities	11 (16.2%)
**MCPyV**	
Not tested	50 (73.5%)
Positive	17 (25%)
Negative	1 (1.5%)
**Tumor stage at diagnosis** (AJCC 2017)	
I	19 (27.9%)
II	24 (35.3%)
III	21 (30.9%)
IV	4 (5.9%)
**Immunosuppression**	
No	54 (79.4%)
Yes	14 (20.6%)
**Treatments**	
None **	35 (51.5%)
Immune checkpoint inhibitors	11 (16.2%)
Chemotherapy	7 (10.3%)
Others ***	15 (22%)
**Outcomes**	
MCC relapse	
No	46 (67.6%)
Yes	22 (32.4%)
Median time to relapse (months) *	12 (3–113)
MCC death	
No	46 (67.6%)
Yes	22 (32.4%)
Median time to death (months) *	18 (3–133)

MCPyV = Merkel cell polyomavirus; * medians and range; ** standard therapy at baseline including surgery and adjuvant radiation; *** definitive radiotherapy, electrochemotherapy, surgery of metastases, etc.

**Table 2:** H-score data of CD200/CD200R protein expression analysis in patients with Merkel cell carcinoma (MCC, *n* = 68).

Parameters	Primary MCC	MCC Metastasis	*p*-Value
**CD200**			
Median (range) H-score	171.5 (3–284)	197 (20–271)	*p* = 0.68
**CD200R**			
Median (range) H-score	167 (53–243)	171 (91–259)	*p* = 0.71

## Data Availability

Derived data supporting the findings of this study are available from the corresponding author, T.G., on reasonable request.

## References

[1] Becker J.C., Beer A.J., DeTemple V.K., Eigentler T., Flaig M., Gambichler T., Grabbe S., Höller U., Klumpp B., Lang S. (2023). S2k Guideline-Merkel cell carcinoma (MCC, neuroendocrine carcinoma of the skin)-Update 2022. J. Der Dtsch. Dermatol. Ges..

[2] Becker J.C., Stang A., DeCaprio J.A., Cerroni L., Lebbé C., Veness M., Nghiem P. (2017). Merkel cell carcinoma. Nat. Rev. Dis. Primers.

[3] Becker J.C., Stang A., Schrama D., Ugurel S. (2024). Merkel Cell Carcinoma: Integrating Epidemiology, Immunology, and Therapeutic Updates. Am. J. Clin. Dermatol..

[4] Stang A., Möller L., Wellmann I., Claaßen K., Kajüter H., Ugurel S., Becker J.C. (2024). Incidence and Relative Survival of Patients with Merkel Cell Carcinoma in North Rhine-Westphalia, Germany, 2008–2021. Cancers.

[5] Nghiem P., Kaufman H.L., Bharmal M., Mahnke L., Phatak H., Becker J.C. (2017). Systematic literature review of efficacy, safety and tolerability outcomes of chemotherapy regimens in patients with metastatic Merkel cell carcinoma. Future Oncol..

[6] Becker J.C., Lorenz E., Ugurel S., Eigentler T.K., Kiecker F., Pföhler C., Kellner I., Meier F., Kähler K., Mohr P. (2017). Evaluation of real-world treatment outcomes in patients with distant metastatic Merkel cell carcinoma following second-line chemotherapy in Europe. Oncotarget.

[7] Angeles C.V., Sabel M.S. (2021). Immunotherapy for Merkel cell carcinoma. J. Surg. Oncol..

[8] Kacew A.J., Dharaneeswaran H., Starrett G.J., Thakuria M., LeBoeuf N.R., Silk A.W., DeCaprio J.A., Hanna G.J. (2020). Predictors of immunotherapy benefit in Merkel cell carcinoma. Oncotarget.

[9] Abu Rached N., Becker J.C., Lonsdorf A.S., Keller A., Zeglis I.A., Gambichler T. (2024). Introducing MCC-PS: A novel prognostic score for Merkel cell carcinoma. Front. Oncol..

[10] Gambichler T., Abu Rached N., Susok L., Becker J.C. (2022). Serum neuron-specific enolase independently predicts outcomes of patients with Merkel cell carcinoma. Br. J. Dermatol..

[11] Guénolé M., Bénigni P., Bourbonne V., Lucia F., Legoupil D., Pradier O., Misery L., Uguen A., Schick U. (2021). The prognostic significance of PD-L1 expression on tumor and immune cells in Merkel cell carcinoma. J. Cancer Res. Clin. Oncol..

[12] Moreaux J., Veyrune J.L., Reme T., De Vos J., Klein B. (2008). CD200: A putative therapeutic target in cancer. Biochem. Biophys. Res. Commun..

[13] Liu J.Q., Hu A., Zhu J., Yu J., Talebian F., Bai X.F. (2020). CD200-CD200R Pathway in the Regulation of Tumor Immune Microenvironment and Immunotherapy. Adv. Exp. Med. Biol..

[14] Lee M.H., Kim Y.J., Yun K.A., Won C.H., Lee M.W., Choi J.H., Chang S.E., Lee W.J. (2020). Prognostic significance of CD200 protein expression and its correlation with COX-2 in cutaneous melanoma. J. Am. Acad. Dermatol..

[15] Talebian F., Liu J.Q., Liu Z., Khattabi M., He Y., Ganju R., Bai X.F. (2012). Melanoma cell expression of CD200 inhibits tumor formation and lung metastasis via inhibition of myeloid cell functions. PLoS ONE.

[16] Talebian F., Yu J., Lynch K., Liu J.Q., Carson W.E., Bai X.F. (2021). CD200 Blockade Modulates Tumor Immune Microenvironment but Fails to Show Efficacy in Inhibiting Tumor Growth in a Murine Model of Melanoma. Front. Cell Dev. Biol..

[17] Petermann K.B., Rozenberg G.I., Zedek D., Groben P., McKinnon K., Buehler C., Kim W.Y., Shields J.M., Penland S., Bear J.E. (2007). CD200 is induced by ERK and is a potential therapeutic target in melanoma. J. Clin. Investig..

[18] Tonks A., Hills R., White P., Rosie B., Mills K.I., Burnett A.K., Darley R.L. (2007). CD200 as a prognostic factor in acute myeloid leukaemia. Leukemia.

[19] Rygiel T.P., Meyaard L. (2011). CD200R signaling in tumor tolerance and inflammation: A delicate balance. Curr. Opin. Immunol..

[20] Erin N., Podnos A., Tanriover G., Duymuş Ö., Cote E., Khatri I., Gorczynski R.M. (2015). Bidirectional effect of CD200 on breast cancer development and metastasis, with ultimate outcome determined by tumor aggressiveness and a cancer-induced inflammatory response. Oncogene.

[21] Su Y., Yamazaki S., Morisue R., Suzuki J., Yoshikawa T., Nakatsura T., Tsuboi M., Ochiai A., Ishii G. (2021). Tumor-Infiltrating T Cells Concurrently Overexpress CD200R with Immune Checkpoints PD-1, CTLA-4, and TIM-3 in Non-Small-Cell Lung Cancer. Pathobiology.

[22] Abu Rached N., Nick M., Susok L., Becker J.C., Gambichler T. (2023). Intra-tumoral CD200/200R protein expression in advanced melanoma patients who underwent treatment with immune checkpoint inhibitors: Preliminary data. J. Dtsch. Dermatol. Ges..

[23] Zheng Y., Tang L., Chen J. (2020). CD200/CD200R axis in cancer immunotherapy: New perspectives on an emerging target. Front. Immunol..

[24] Gaiser M.R., Weis C.A., Gaiser T., Jiang H., Buder-Bakhaya K., Herpel E., Warth A., Xiao Y., Miao L., Brownell I. (2018). Merkel cell carcinoma expresses the immunoregulatory ligand CD200 and induces immunosuppressive macrophages and regulatory T cells. Oncoimmunology.

[25] Love J.E., Thompson K., Kilgore M.R., Westerhoff M., Murphy C.E., Papanicolau-Sengos A., McCormick K.A., Shankaran V., Vandeven N., Miller F. (2017). CD200 Expression in Neuroendocrine Neoplasms. Am. J. Clin. Pathol..

[26] American Joint Committee on Cancer (2017). AJCC Cancer Staging Handbook. Merkel Cell Carcinoma.

[27] Wieland U., Mauch C., Kreuter A., Krieg T., Pfister H. (2009). Merkel cell polyomavirus DNA in persons without merkel cell carcinoma. Emerg. Infect. Dis..

[28] Gambichler T., Dreißigacker M., Kasakovski D., Skrygan M., Wieland U., Silling S., Gravemeyer J., Melior A., Cherouny A., Stücker M. (2021). Patched 1 expression in Merkel cell carcinoma. J. Dermatol..

[29] Bankhead P., Loughrey M.B., Fernández J.A., Dombrowski Y., McArt D.G., Dunne P.D., McQuaid S., Gray R.T., Murray L.J., Coleman H.G. (2017). QuPath: Open source software for digital pathology image analysis. Sci. Rep..

[30] Gambichler T., Gnielka M., Rüddel I., Stockfleth E., Stücker M., Schmitz L. (2017). Expression of PD-L1 in keratoacanthoma and different stages of progression in cutaneous squamous cell carcinoma. Cancer Immunol. Immunother..

[31] Khan I.Z., Del Guzzo C.A., Shao A., Cho J., Du R., Cohen A.O., Owens D.M. (2021). The CD200-CD200R Axis Promotes Squamous Cell Carcinoma Metastasis via Regulation of Cathepsin, K. Cancer Res..

[32] Liu J.Q., Talebian F., Wu L., Liu Z., Li M.S., Wu L., Zhu J., Markowitz J., Carson WE 3rd Basu S., Bai X.F. (2016). A Critical Role for CD200R Signaling in Limiting the Growth and Metastasis of CD200+ Melanoma. J. Immunol..

[33] Nip C., Wang L., Liu C. (2023). CD200/CD200R: Bidirectional Role in Cancer Progression and Immunotherapy. Biomedicines.

[34] Lin T.L., Lu C.T., Karmakar R., Nampalley K., Mukundan A., Hsiao Y.-P., Hsieh S.-C., Wang H.-C. (2024). Assessing the efficacy of the spectrum-aided vision enhancer (SAVE) to detect acral lentiginous melanoma, melanoma in situ, nodular melanoma, and superficial spreading melanoma. Diagnostics.

[35] Huang H.-Y., Hsiao Y.-P., Mukundan A., Tsao Y.-M., Chang W.Y., Wang H.-C. (2023). Classification of skin cancer using novel hyperspectral imaging engineering via YOLOv5. J. Clin. Med..

